# The effects of pain on quadriceps strength, joint proprioception and dynamic balance among women aged 65 to 75 years with knee osteoarthritis

**DOI:** 10.1186/s12877-018-0932-y

**Published:** 2018-10-17

**Authors:** Dokyung Kim, Geon Park, Liang-Tseng Kuo, Wonhah Park

**Affiliations:** 1 Department of Sports Medicine Center, Samsung Medical Center, Sungkyunkwan University School of Medicine, 81 Irwon-ro, Gangnam-gu, Seoul, 135-710 South Korea; 2 Sports Medicine Center, Department of Orthopaedic Surgery, Chang Gung Memorial Hospital, No. 6 West Sec, Chia-Pu Road, Putz city, Chiayi, 613 Taiwan; 3 Chang Gung University of Science and Technology, Chiayi, Taiwan

**Keywords:** Knee osteoarthritis, Isokinetic knee strength, Pain, Proprioception, Dynamic balance

## Abstract

**Background:**

Patients with knee osteoarthritis (OA) were reported to have quadriceps weakness, and impaired proprioception, both related to pain and swelling. It is unclear whether pain alone a causal factor to above findings over the knee joint. The purpose of this study was to assess the effects of knee pain alone on the quadriceps strength, proprioception and dynamic balance in subjects with bilateral knee OA without joint swelling.

**Methods:**

Fourty females with mean age of 68.3 years were involved in this cross-sectional study. The inclusion criteria were bilateral knee OA without joint swelling, with a visual analogue pain scale difference (> 1) between each knee. Patients all underwent assessment of the isokinetic strength of knee muscles, knee proprioceptive acuity, and dynamic balance.

**Results:**

Patients’ more painful knee had weaker isokinetic quadriceps strength than less painful knee at both 60 °/s and 180 °/s (*p* = 0.01, *p* = 0.01, respectively). There were no differences in proprioceptive acuity between both knees in all three knee positions. Meanwhile, there was a significant difference in the dynamic balance index measurement between both knees (more painful versus less painful: 3.88 ± 1.15 vs. 3.30 ± 1.00, *p* = 0.01). Quadriceps strength was associated with dynamic balance stability (60 °/s, *r* = − 0.578, *p* <  0.01; 180 °/s, *r* = − 0.439, *p* <  0.01).

**Conclusions:**

For patients with knee OA, the more painful knee was associated with weaker quadriceps and poor balance ability. To improve lower limb function and balance stability of the older persons having knee OA, physicians should take the optimal pain management strategy.

**Electronic supplementary material:**

The online version of this article (10.1186/s12877-018-0932-y) contains supplementary material, which is available to authorized users.

## Background

Knee osteoarthritis (OA) is a chronic disease that manifests as pain, stiffness, swelling and muscle weakness [1, 2], causes functional impairment due to lower limb muscle weakness, and is one of the most common diseases in the older persons [3–7]. A previous study reported that subjects with knee pain had a significantly higher incidence of quadriceps weakness after a 30-month period than subjects without pain and that patients with knee OA had a 76% reduction in eccentric quadriceps strength than healthy subjects [8]. Meanwhile, quadriceps muscle weakness may contribute to worsening of knee pain [9]. Also, quadriceps muscle strength is one of the intrinsic factors affecting the knee functions [1, 5].

Furthermore, patients with knee OA have impaired proprioception when compared to healthy people. Some studies described impaired proprioception as associated with the presence of pain [4], and knee pain was a significant predictor of decrease in balance. Also, the studies found that pain had effects, not only on muscle strength but also on proprioception [10, 11].

In patients with knee OA, decreased muscle strength and proprioception are known to be affected by swelling as well as pain. Swelling is known to trigger the spinal inhibitory mechanism of quadriceps motor neurons, and to reduce muscle activity, thereby decreasing proprioception [12, 13]. However, there are varied opinions on the effects of knee pain alone on quadriceps muscle weakness, namely decreased proprioception and balance stability in patients with knee OA, without swelling [14, 15]. It is unclear whether such functional impairments in patients with knee OA are due to swelling, or affected by the degree of pain, as related studies are scarce. There are also a few studies that designed to compare those results for each knee pain stage in OA and healthy subjects. Therefore, in this study, we aimed to investigate patients with knee OA without swelling that had different degrees of pain in each side of knee joints.

The purpose of this study was to investigate the effects of knee pain on quadriceps muscle strength, knee joint proprioception, and dynamic balance stability. We planned to compare outcomes between each knee in the same patient with knee OA. We also investigate the correlation between quadriceps muscle strength, knee joint proprioception, and dynamic balance stability.

## Methods

### Subjects

We conducted a cross-sectional study. Patients who visited our clinic due to knee pain were evaluated. Patients who met the following criteria were selected for the study:

1) Female patients, aged between 65 and 75 years, who had a diagnosis of knee OA according to the American College of Rheumatology criteria [16];

2) both knee joints with arthritic change grade 2 or higher in the Kellgren-Lawrence grading scale [17];

3) both knees have pain without swelling confirmed by ultrasonography, which performed by a senior radiologist who had performed ultrasonography for more than five years; and.

4) the difference of visual analogue scale (VAS) greater than one point between each knee joint.

The exclusion criteria were polyarthritis, the presence of rheumatoid arthritis or other systemic inflammatory arthropathies, any lower limb surgery within the last 12 months or a history of knee arthroplasty surgery, intraarticular corticosteroid injections into either knee in the previous month. Participants were also excluded if they had a neurologic or musculoskeletal condition that affected their balance or movement (i.e., Parkinson disease, multiple sclerosis).

All participants provided informed consent before testing, and the study was approved by the Sungkyunkwan University School of Medicine Clinical Research Ethics Board (SMC 2017–05–084-001). An a priori power analysis was performed to determine the sample size using a two-sided hypothesis test at an alpha level of 0.05 and a power of 0.8. The results of a previous study indicated that 25 knees would be required to detect a significant between-group joint proprioception difference of > 1°, the primary outcome measure [18]. The reporting of this study conforms to the STROBE statement [19].

### Measures

Each subject had X-ray radiographs of both knees and completed pain evaluation as part of the study. Using the VAS scale, a score between 0 to 10 points of the combined pain in both knees was obtained, followed by separate scores for each knee, to identify the more and less painful. At the same time the quadriceps muscle strength, knee joints proprioception, and balance tests were performed after approximately 10 min of warm-up consisting of the stretches to hamstring, quadriceps, and calf muscles, and then a light level of stationary bike exercise. All observations and measurements were carried out by the same investigator.

### Knee muscle strength

The strength and endurance of the knee extensor and flexor muscles were evaluated using an isokinetic dynamometer (CSMI Medical Solutions, MA, USA). The maximal strength of the quadriceps and hamstring was measured for each knee at 60 °/s (4 repetitions) and 180 °/s (20 repetitions), and mean power for each was calculated [20, 21]. The maximum peak torque (Nm) for each velocity was also recorded. This measure has excellent intra-rater reliability in patients with knee OA [22].

### Joint proprioception

Knee joint proprioception was assessed using a Biodex System 3 (Biodex Medical, Shirley, NY, USA) and a knee joint repositioning task, previously used in those with knee OA to assess joint proprioception [23, 24]. Subjects were seated and blindfolded for the task. The leg was passively positioned and held for 10 s in 1 of 3 target positions (15°, 30°, and 45° of knee flexion). The knee was then returned to 90° knee flexion, and the subject was asked to match the position their limb had been held. Two blocked trials at each of the three randomly presented target positions were completed, resulting in a total of six trials. Each trial was calculated with the absolute difference between actual leg position and target position degrees.

### Dynamic balance stability

Dynamic Balance stability was measured with the Biodex Stability System (BSS, Biodex Medical Systems, Shirley, NY, USA). The reliability of this measurement was confirmed in a study by Wikstrom et al. [25]. The balance platform was set to tilt to all directions. All participants were trained one minute for adaptation to the machine, following which three practice trials, to reduce any learning effects, and three test evaluations were performed in each measurement session. The stability index represents the variance of platform displacement in degrees from level. The participant’s ability to control the angle of tilt on the platform was measured by the system. The higher number of the index meant more motion, which indicates a problem with balance [26]. A mean score of stability index was calculated from the three trials. Between each section, they were allowed to take a break for three minutes to minimize the effects of muscles fatigue on the results. The single-leg balance ability index was measured by overall stability score, which was reported to the best indicator of the dynamic balance ability [27].

### Statistical analysis

Statistical analysis was performed using SPSS version 22 (SPSS Inc., Chicago, IL, USA) and MedCalc for Windows, version 15.0 (MedCalc Software, Ostend, Belgium). Normality of all data was checked using the Shpiro-Wilk test (*p* > 0.05). To investigate the effect of pain on muscle strength, proprioception, and dynamic balance, each knee of the subjects was grouped by the intensity of pain (more painful knee versus less painful knee). Paired t-test was used to compare the difference in muscle strength, joint proprioception, and dynamic balance ability between the two groups. Pearson’s correlation coefficients were used to test the correlation between the muscle strength at a different angle of velocities and the other two parameters including knee joint proprioception and dynamic balance ability. Only when the coefficient is more than 0.7 or minus than − 0.7, the relationship between variates is considered strong. A backward multiple regression analysis was performed to calculate the coefficient of factors affecting the dynamic balance. Statistical significance was accepted at a *p* <  0.05.

## Results

There was the total of 40 female patients in this study. The detail of the demographic data was shown in Table 1.

**Table 1:** Demographics and characteristics of study subjects (*N* = 40)

Characteristics	Mean ± SD
Age (years)	68.3 ± 3.2
Height (cm)	153.0 ± 5.0
Body mass (kg)	53.1 ± 6.1
Body mass index (kg/m^2^)	22.6 ± 2.6
Dominant leg (right/left)	33/7
More painful knee (right/left)	29/11
Visual analogue scale	
More painful knee	5.90 ± 1.39
Less painful knee	3.52 ± 1.37

Values were presented as mean ± standard deviation

### Effect of knee pain on knee strength, proprioception, and dynamic balance

With respect to knee extensor muscle strength, the more painful knees showed significantly lower strength than the less painful knees at angular velocities of both 60 °/s (54.98 ± 13.36 Nm vs. 63.00 ± 9.96 Nm, *p* = 0.01) and 180°/s (37.78 ± 9.55 Nm vs 44.68 ± 11.77 Nm, *p* = 0.01). However, there was no statistically significant difference in knee flexor muscle strength at angular velocities of 60 °/s or 180 °/s either (Table 2).

**Table 2:** Isokinetic knee extensor strength

Isokinetic peak torques (Nm)	More painful site	Less painful site	*p* value
Extensor 60 °/sec	54.98 ± 13.36	63.00 ± 9.96	0.01*
Extensor 180 °/sec	37.78 ± 9.55	44.68 ± 11.77	0.01*
Flexor 60 °/sec	27.10 ± 6.70	29.88 ± 6.57	0.06
Flexor 180 °/sec	23.30 ± 7.25	25.50 ± 5.61	0.07

Values were presented as mean ± standard deviation

*p < 0.05

The proprioception of the knee joint was measured at 15°, 30°, and 45° of knee flexion using the knee joint reposition tests. We found no statistically significant difference in the proprioception measurements between the knees (Table 3). However, the dynamic balance index measurement showed a significant difference between the more and less painful knees (3.88 ± 1.15 vs. 3.30 ± 1.00, *p* = 0.01).

**Table 3:** Joint proprioception and balance index^a^

Test	More painful site	Less painful site	*p* value
Joint proprioception^b^			
15° knee flexion	4.50 ± 1.00	4.36 ± 0.89	0.27
30° knee flexion	4.25 ± 1.02	4.22 ± 10.71	0.89
45° knee flexion	4.12 ± 0.79	4.03 ± 1.06	0.65
Dynamic balance index^c^	3.88 ± 1.15	3.30 ± 1.00	0.01*

^a^Values were presented as mean ± standard deviation

^b^Absolute average error in degrees

^c^The higher values meant the poor balance ability

*p < 0.05

### Correlation between quadriceps strength, proprioception ability, and dynamic balance measurements

There was no significant correlation between extensor muscle strength and proprioception (all *p* > 0.05, shown in Table 4). There were significant associations between dynamic balance measurements and quadriceps strength at angular velocity of 60 °/s and 180 °/s (*r* = − 0.578, *p* <  0.01; *r* = − 0.439, *p* <  0.01, respectively. Table 4, Additional file 1: Figure S1, Additional file 2: Figure S2). Multiple regression analysis showed that dynamic balance was significantly influenced by quadriceps muscle strength, either at angular velocity of 60 °/s or 180 °/s (coefficient = − 0.047, *p* <  0.01; coefficient = − 0.032, *p* <  0.01, Table 5).

**Table 4:** Correlation between muscle strength, joint proprioception and dynamic balance

	Quadriceps muscle strength			
	at 60 °/sec		at 180 °/sec	
	Correlation (r)^a^	*p* value	Correlation (r)^a^	*p* value
Joint proprioception				
15° Knee flexion	- 0.051	0.75	- 0.106	0.51
30° knee flexion	- 0.044	0.78	- 0.047	0.77
45° knee flexion	0.177	0.27	0.071	0.66
Dynamic balance	- 0.578	< 0.01*	- 0.439	< 0.01*

^a^Pearson’s correlation coefficient

*p < 0.05

**Table 5:** Correlation between muscle strength, joint proprioception, pain scale, and dynamic balance index

Independent variables	Coefficient	Standard error	r_partial_	*p* value
Model 1				
Quadriceps strength at 60°/s	−0.047	0.009	−0.506	< 0.01*
Proprioception (knee flexion 30°)	0.214	0.116	0.208	0.07
Pain (VAS)	0.079	0.062	0.144	0.21
Model 1				
Quadriceps strength at 180°/s	−0.032	0.011	−0.308	< 0.01*
Proprioception (knee flexion 30°)	0.162	0.127	0.145	0.21
Pain (VAS)	0.132	0.069	0.213	0.06

VAS, visual analogues scale

*p < 0.05

## Discussion

The principal findings of this study are that patients had less quadriceps muscle strength at angular velocities of both 60 °/s and 180 °/s, poor dynamic balance stability, but similar proprioceptive acuity in their more painful knees while comparing to their less painful knees. For the association between these factors, quadriceps strength is not correlated with proprioceptive acuity but moderately correlated with dynamic balance stability. That is, OA patients had weaker quadriceps muscle strength and poor dynamic balance stability in the more painful knees.

For clinical assessment of lower limb mobility in the older persons, lots of modalities were discussed in previous studies. Ward et al. used strength (leg strength), speed of movement (leg velocity, reaction time, rapid leg coordination), range of motion (ROM) (knee flexion/knee extension/ankle ROM), asymmetry (asymmetry of leg strength and knee flexion/extension ROM measures), and trunk stability (trunk extensor endurance, kyphosis) to assess the lower limb mobility in the older persons [28]. They found that baseline weaker leg strength, trunk extensor endurance, and slower leg velocity predicting a higher likelihood of persistently poor function over two years. Baseline weaker leg strength, trunk extensor endurance, and restricted knee flexion motion also predicted a higher likelihood of a decline in function [28]. According to a recent systematic review, people with knee OA had inferior performance than healthy controls on Step Test, Single-Leg Stance Test, Functional Reach Test, Tandem Stance Test, and Community Balance and Mobility Scale [29]. In our present study, we used knee muscle strength measured in isokinetic dynamometer and dynamic balance test to evaluate the lower limb function in our subjects.

Concerning the effects of knee pain on femoral muscle strength, Messier et al. [8] reported that subjects with chronic painful knee had the significant decline in quadriceps muscle strength after a 30-month period, compared with those without knee pain. Hassan et al. [11] reported that in patients with knee osteoarthritis, there was no difference in knee joint proprioception and balance stability, but quadriceps muscle strength was increased after reducing pain by local anesthetic injection into the knee joint. Also, quadriceps muscle weakness contributes to pain and physical disability in patients with knee OA [14, 15], and the previous studies also showed that knee extensor strength was significantly correlated with pain, stiffness, and function in patients with knee OA [30]. A large-scale population-based cohort study also showed that quadriceps muscle strength was independently associated with knee pain regardless of the radiologic stage of knee OA [31].

In this study, we investigated the effects of pain on quadriceps muscle strength in patients with knee OA by comparing the strength between the more painful knee and the less painful knee in each subject. Results showed there was a significant difference in knee extensor muscle strength between the knees, but there was no difference in the knee flexor muscle strength. These results suggest that knee pain was associated with quadriceps strength weakness, and the cause may be reflex arthrogenous muscle inhibition of quadriceps caused by pain during the muscle strength measurement. This mechanism was thought to be due to the selective inactivation or weakening of quadriceps muscle by knee pain or other causes (tender point, anxiety) in patients with knee arthropathy, as found in previous studies [32, 33].

Previous studies reported conflicting results concerning the effects of knee pain on proprioception. Felson et al. [34] reported the correlation between impaired knee joint proprioception and the presence and severity of knee pain, but Hall et al. [35] reported that there was no significant difference in knee joint proprioception between the more painful knee and the less painful knee within the same patient. The finding of our study also supported Hall’s study. The reasons why the results of studies on the effects of pain on proprioception were inconsistent are thought to be mainly due to the following two factors. Firstly, the Multicenter Osteoarthritis Study investigated not only subjects with knee OA but also those at high risk of developing OA including obesity, patients with a history of knee injury and patients with a history of knee surgery [36]. Overweight and knee surgery both lead to muscles weakness and the decline in proprioception ability, which may lead to an inevitable bias to the findings [37]. Secondly, the method used for evaluating knee pain in our study was different from the previous studies [34, 35]. In previous studies, the pain intensity was evaluated by participants’ subjective feeling in the knees in the previous month, which was simply classified as ‘yes’ or ‘no’. In contrast, our study involved patients who had no knee swelling and had bilateral knee pain with a VAS difference between the knees of more than two points, to ensure more reliable results were obtained.

Our study found that there were differences in balance stability between patients’ more painful knee and less painful knee. Hassan et al. [11] reported that quadriceps muscle strength was significantly improved but not proprioception and balance stability after pain relief by intraarticular anesthetic injection. That is, pain level did not influence the dynamic balance stability, which is different from the finding of our study. Balance stability is a complex process that is maintained through coordinated neuromuscular reaction by the central nervous system (CNS) [38, 39]. This also includes sensory processing information transmitted from the vestibular organ, visual and somatic sensory organs, and involves multiple factors such as knee joint proprioception, femoral muscle strength, visual and CNS reaction. Therefore, it is difficult to consider the effects of pain in isolation. However, the quadriceps muscle is the principal dynamic stabilizer of the knee joint; thus, quadriceps muscle weakness leads to instability of the knee, which may be one of the reasons for balance instability and knee pain.

This study encountered several limitations. First, the study had a relatively small number of subjects with the cross-sectional test. Thought we enrolled 40 participants in the current study, the power was still insufficient to detect the difference in primary outcomes (post-hoc analysis, power = 0.255). Second, this study comprised only females aged 65 to 75 years, which limited the generalisability of this study. Third, our study did not take into account the medication patients had. However, the effects of medications are equal to both knees, and our study only included the patients with VAS scores differences of greater than two points between each knee joint, which made our study to compare the outcomes between both knees possible. Fourth, this study selected both knee OA patients with different pain intensity in each knee (VAS > 1) to test the effect of pain on knee outcomes. However, no current study was available to support or against the criteria for the difference of pain score. The future study with larger sample size, enrolling male gender will be needed to validate the findings of this study.

## Conclusion

In aged women with knee OA, knee pain was associated with weak quadriceps strength and poor balance ability. Thus, the pain management strategy for the older persons with knee OA should be optimized to improve lower limb function and balance stability.

## Additional files

Additional file 1: **Table S1.** Association between balance index and quadriceps strength at 60 °/s. The balance index was significantly associated with quadriceps strength at 60°/s (*p* <  0.01). (TIF 49 kb)

Additional file 2: **Table S2.** Association between balance index and quadriceps strength at 180 °/s. The balance index was significantly associated with quadriceps strength at 180°/s (*p* < 0.01). (TIF 47 kb)

## Abbreviations

CNS: Central nervous system
OA: Osteoarthritis
ROM: Range of motion
VAS: Visual analogue scale
