# Supplementary figures and images for: The effects of pain on quadriceps strength, joint proprioception and dynamic balance among women aged 65 to 75 years with knee osteoarthritis
Source: BMC Geriatr. 2018 Oct 17;18:245. doi: 10.1186/s12877-018-0932-y (PMC6192068; doi:10.1186/s12877-018-0932-y)

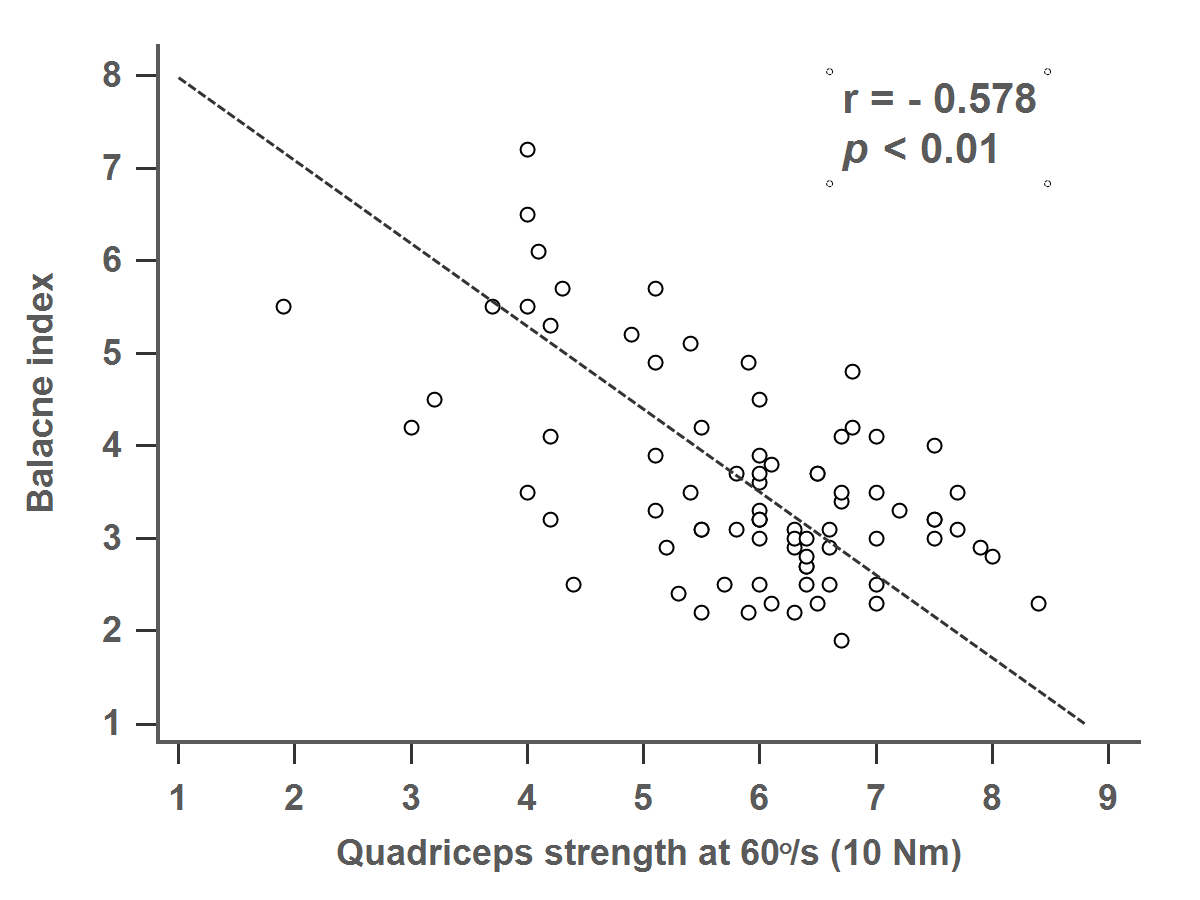

Supplement: Supplementary file 1 — Table S1. Association between balance index and quadriceps strength at 60 °/s. The balance index was significantly associated with quadriceps strength at 60°/s (p < 0.01). (TIF 49 kb) [file 12877_2018_932_MOESM1_ESM.tif]

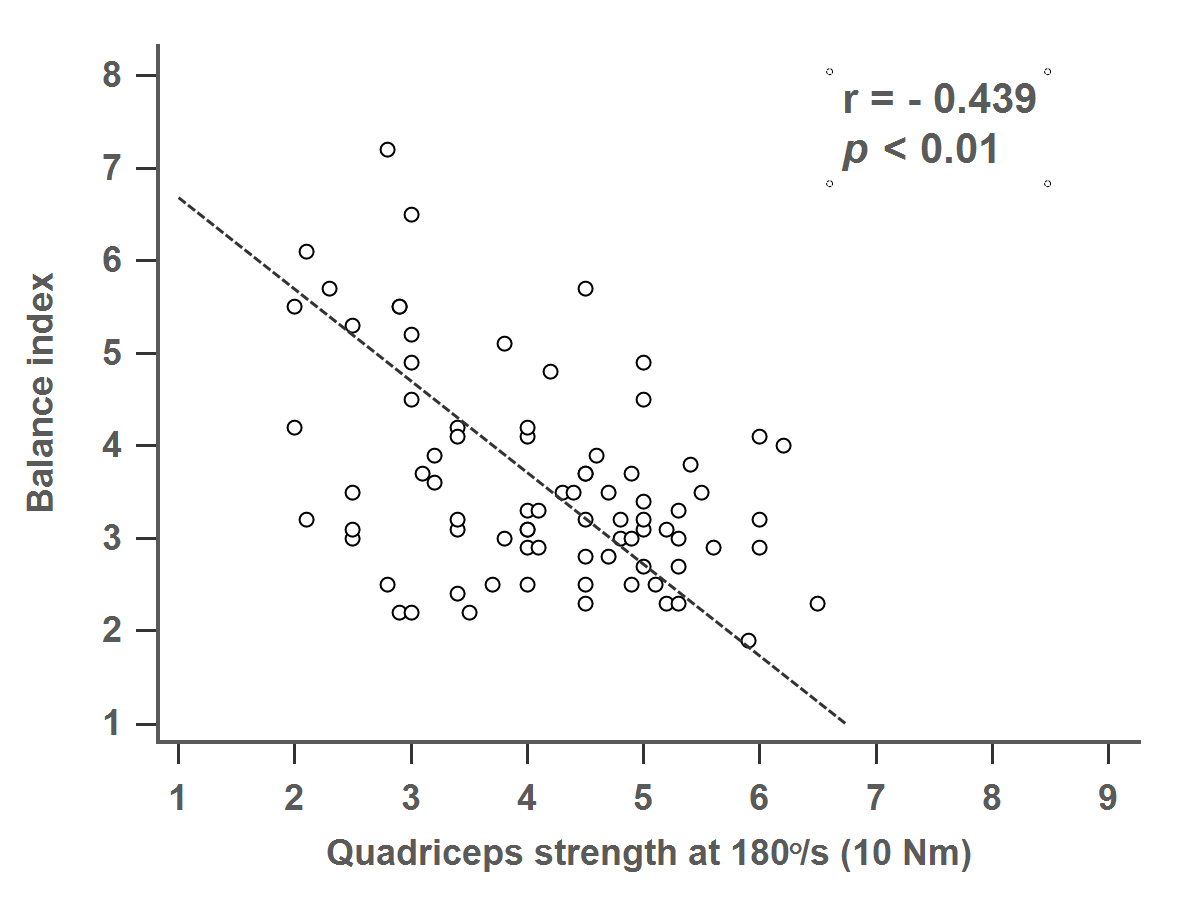

Supplement: Supplementary file 2 — Table S2. Association between balance index and quadriceps strength at 180 °/s. The balance index was significantly associated with quadriceps strength at 180°/s (p < 0.01). (TIF 47 kb) [file 12877_2018_932_MOESM2_ESM.tif]
